# Medicaid managed care: how to target efforts to reduce costs

**DOI:** 10.1186/1472-6963-14-461

**Published:** 2014-11-14

**Authors:** Mary E Charlson, Martin T Wells, Balavenkatesh Kanna, Van Dunn, Walid Michelen

**Affiliations:** Division of Clinical Epidemiology and Evaluative Sciences Research and Center for Integrative Medicine at Weill Cornell Medical College, New York, USA; Department of Statistical Science, Cornell University, Ithaca, NY USA; Gotham Health, NYC Health and Hospitals Corporation, New York, USA; MetroPlus Health Plan, New York City Health and Hospitals Corporation, New York, USA; Center for Integrative Medicine, Weill Cornell Medical College, 1300 York Avenue, Box 46, New York, NY 10065 USA

**Keywords:** Comorbidity, Multiple chronic conditions, Medicaid, Prediction of cost, Predictive models, Cost containment, Managed care, Cost analysis, Prediction rules

## Abstract

**Background:**

To be successful, cost control efforts must target Medicaid Managed Care (MMC) beneficiaries likely to incur high costs. The critical question is how to identify potential high cost beneficiaries with simple, reproducible, transparent, auditable criteria. Our objective in this analysis was to evaluate whether the total burden of comorbidity, assessed by the Charlson comorbidity index, could identify MMC beneficiaries who incurred high health care costs.

**Methods:**

The MetroPlus MMC claims database was use to analyze six months of claims data from 07/07-12/07; the analysis focused on the total amount paid. Age, gender, Charlson comorbidity score, serious mental illness and pregnancy were analyzed as predictors of total costs.

**Results:**

We evaluated the cost profile of 4,614 beneficiaries enrolled at MetroPlus, an MMC plan. As hypothesized, the comorbidity index was a key correlate of total costs (p < .01). Yearly costs were more related to the total burden of comorbidity than any specific comorbid disease. For adults, in addition to comorbidity (p < .01) both serious mental illness (p < .01) and pregnancy (p < .01) were also related to total costs, while age, drug addiction and gender were not. The model with age, gender, comorbidity, serious mental illness, pregnancy and addiction explained 20% of the variance in total costs. In children, comorbidity (p < .01), serious mental illness (p < .01), addiction (p < .03) and pregnancy (p < .01) were associated with log cost; the model with those variables explained 6% of the variance in costs.

**Conclusions:**

Comorbidity can be used to identify MMC beneficiaries most likely to have high costs.

## Background

States have steadily increased mandates for Medicaid recipients to enroll in Medicaid Managed Care (MMC). As a result, 70% of the 60 million Medicaid beneficiaries are now in a MMC plan [1]. While some specific MMC programs have shown potential cost savings primarily by reducing inpatient use [2], an analysis of the 1991-2003 experience with MMC mandates in 50 states found that their impact on overall costs was negligible [3].

It has been estimated that under the Patient Protection and Affordable Care Act 25%, of U.S. residents are likely to be insured by Medicaid [4], increasing the need to identify potential high cost MMC beneficiaries with simple, reproducible, and auditable criteria that can be applied prospectively as patients enroll.

Previously among primary care patients, we found that average yearly total costs rose exponentially as the Charlson comorbidity rose above 4 [5]. However, the weakness of this prior study was that our cost data captured only costs of primary care patients at one health center (i.e., New York Presbyterian), and not outpatient or inpatient costs for external providers [5]. The Charlson comorbidity index can be assessed by questionnaire, analysis of medical records or claims data [6, 7].

In this analysis of beneficiaries enrolled in an MMC plan, our objective was to evaluate whether the comorbidity index identified Medicaid beneficiaries who incurred high health care costs, controlling for other important contributors to costs in Medicaid patients, such as mental illness and pregnancy [8]. Based on our previous work, we hypothesized that as the comorbidity index rose above 4, there would be an exponential rise in mean yearly cost. If so, the comorbidity index, which can be assessed during a short interview, could be used to identify patients likely to incur high costs at the time of enrollment including those who will be newly enrolled under the Affordable Care Act.

### The population

Lincoln Medical and Mental Health Center (Lincoln), serves the 654,360 predominately Latino (69%) and African American (29%) residents of the South Bronx, who have a median household income of $16,000. Lincoln Medical and Mental Health Center, is part of New York City’s Health and Hospitals Corporation (HHC) - a part owner of Metro Plus which is an MMC plan that assumes risk on all patients assigned to its primary care physicians. The study population includes all enrollees in the MMC plan who have primary care in the Lincoln system, including those enrolled through Medicaid, Child Health Plus, Family Health Plus, and Medicaid HIV. It does not include Medicare/Medicaid patients.

The project was reviewed and approved by the IRB at Lincoln Medical and Mental Health Center, at Weill Cornell Medical College and the HHC central research office. All data was de-identified. For research purposes, the data provided by Metroplus was restricted only to patients who had an assigned primary care physician at Lincoln.

### Sample

The MetroPlus claims database was used to identify those patients for whom Lincoln was assigned as the primary care provider. The analysis focused on patients who had an assigned primary care provider at Lincoln between 07/07-12/07. There were 4,614 MetroPlus members who met the criteria: 2,218 adults and 2,396 children.

### Study variables

The age, gender, primary language, line of service (Medicaid, Child Health Plus, Family Health Plus, Medicaid HIV), and zip code of residence were documented. Chronic diseases were ascertained from the ICD-9 codes assigned by MetroPlus MMC when patients received any service.

The Charlson comorbidity score was used to assess the aggregate burden of chronic disease [9]. The index assigns weights for specific diseases and the total score is calculated by adding the weights [6]. The 23 chronic diseases (including one medication) that comprise the Charlson Comorbidity Index and their assigned weights are as follows:

**Weight of one:** cerebrovascular disease, congestive heart failure, connective tissue disease, chronic obstructive pulmonary disease, dementia, depression, diabetes, hypertension, mild liver disease, myocardial infarction, peripheral vascular, ulcer disease or the use of warfarin;

**Weight of two:** hemiplegia, moderate/severe renal disease, diabetes with end organ damage, any tumor, leukemia, lymphoma or skin ulcers/cellulitis;

**Weight of three:** moderate to severe liver disease;

**Weight of 6:** metastatic solid tumor or AIDS.

Aggregate ICD-9 codes were used to ascertain the Charlson index using the Deyo coding strategy [7]. In addition to chronic conditions that are included in the comorbidity index, substance abuse, mental illness and pregnancy were also recorded. Patients with ICD-9 codes of 303 or 305 were coded as addiction (drug or alcohol) and those with ICD-9 codes of 290, 300 or 307-317 as serious mental illness. Patients with ICD-9 codes of 640-677 were classified as pregnant.

### Costs

Six months of claims data from 07/07-12/07 from MetroPlus MMC costs were evaluated. Costs were assessed by setting and type. The MMC covered inpatient, outpatient, ER, laboratory tests, and prescription drugs; it did not cover dental services. Total costs were defined as the total claims paid; claims that were not paid did not contribute to total costs.

### Statistical analysis

Adjusted costs for patients with specific chronic diseases were analyzed in order to document the cost of that disease alone, controlling for comorbidity. The adjusted comorbidity index was found by subtracting the weight for each disease from the comorbidity index; an index of zero means the patient had just one chronic disease. For example, when evaluating costs associated with diabetes, a patient with only diabetes ( and a comorbidity score of 1) would have an adjusted comorbidity score of 0; however, a patient with diabetes and hypertension would have an adjusted comorbidity score of 1.

*A two part regression* modelling framework was used for modeling log total health care cost. Log total costs are used in the regression modeling in order to correct skewness. The first part of the two-part model is a binary outcome model that describes the distinction between non-users (zero cost) and users of services (non-zero cost), while the second part is a linear regression that describes the distribution of log total health care cost for patients who used services. Separate (two part) regression models were developed for adults and children (age < 18). These regressions were all controlled for age and gender. Addiction (drug or alcohol) and pregnancy were also assessed in both the adult and child models [10].

*Quantile regression* was employed to assess the relationship between predictors and the upper 5% and 10% of the cost distribution, controlling for age, gender and mental health diagnoses [11]. Quantile regression focuses on the upper tail of the cost distribution so that patients with zero costs in the lower tail of the distribution do not heavily affect the estimates. The pseudo R^2^ is the measure of model fit.

## Results

Overall, as shown in Table 1, 48.1% of the MMC beneficiaries were adults, 72% women, with an average age of 45.7 ± 12.6 years (a range of 18 to 73 years), while 51.9% were children with an average age of 7.6 ± 4.9 years (a range of .01 to 17.9 years). Of the adults, 1.8% were over 65 years of age. Of the children, 4.4% were less than one year of age. Overall, 86.9% of all children were enrolled through standard Medicaid, 2.2% through Child Health Plus, a NY State insurance for children under the age of 19 who are not eligible for Medicaid; 8.9% through Family Health Plus, a NY State plan for families not eligible for Medicaid; 0.7% through a commercial MetroPlus plan; and 1.3%, through a Medicaid HIV special needs plan. There were only 8 patients who were enrolled in Medicaid at the time of hospitalization. English was the primary language for 62% and Spanish for 36% of the patients.

**Table 1:** **Demographic and clinical characteristics of beneficiaries**

	Adults (n = 2,218)	Children (n = 2,396)
**Age ( sd)**	45.7 ± 12.6	7.6 ± 4.9
**Female**	71.1%	49.2%
**Medicaid**	78.2%	95.0%
**Child health plus**	0%	4.3%
**Family health plus**	18.5%	0%
**Pregnancy**	4.2%	0.2%
**Addiction**	4.2%	0.3%
**Mental health**	7.4%	1.1%
**Hypertension**	37.2%	0.4%
**Diabetes** ^**€**^	21.4%	0.5%
**Cancer** ^**∞**^	4.1%	0.3%
**Asthma or COPD**	15.2%	11.4%
**Depression**	7.1%	0.3%
**Liver disease** ^**£**^	3.7%	0.1%
**HIV/AIDS**	2.6%	0.4%

^∞^0.2% had metastatic disease.

^€^2.3% had end organ damage.

^£^2.9% with moderate-severe disease.

Table 2 shows the distribution of beneficiaries and costs according to the comorbidity index. Overall, the 8.3% of adults with a comorbidity index ≥4 incurred 30.1% of the costs. Among the children, a vast majority had a comorbidity score of 0. Among children, the 12.7% with one or more chronic illnesses incurred 41.6% of the costs. The most common chronic diseases in children were asthma (11.3%) and diabetes (0.4%). The children with asthma had mean costs of $1,291 ± $7,683, while those with diabetes had mean costs of $2,031 ± $2,013, respectively. Costs did not differ according to enrollment plan, except for Medicaid HIV, where the costs were significantly higher (p < .04).

**Figure 1:**
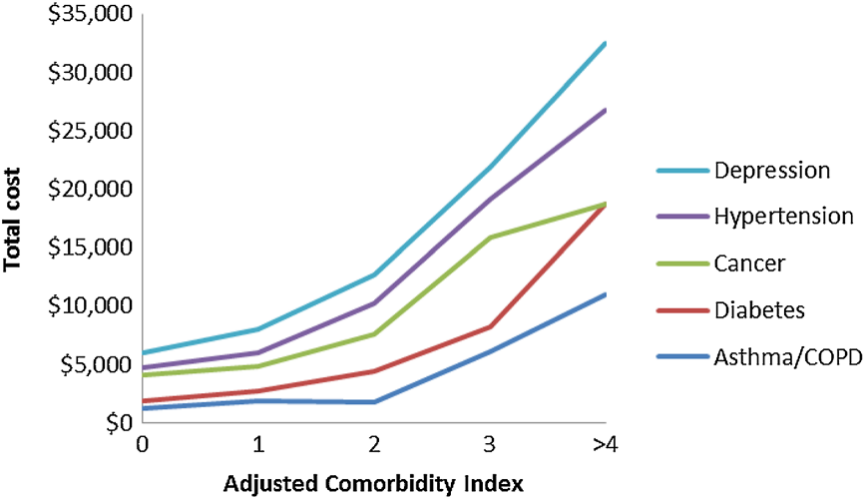
**Costs according to specific chronic diseases and adjusted comorbidity score.** The adjusted comorbidity index was found by subtracting the weight for each disease from the comorbidity index; an index of zero means the patient had just the one chronic disease.

**Figure 2:**
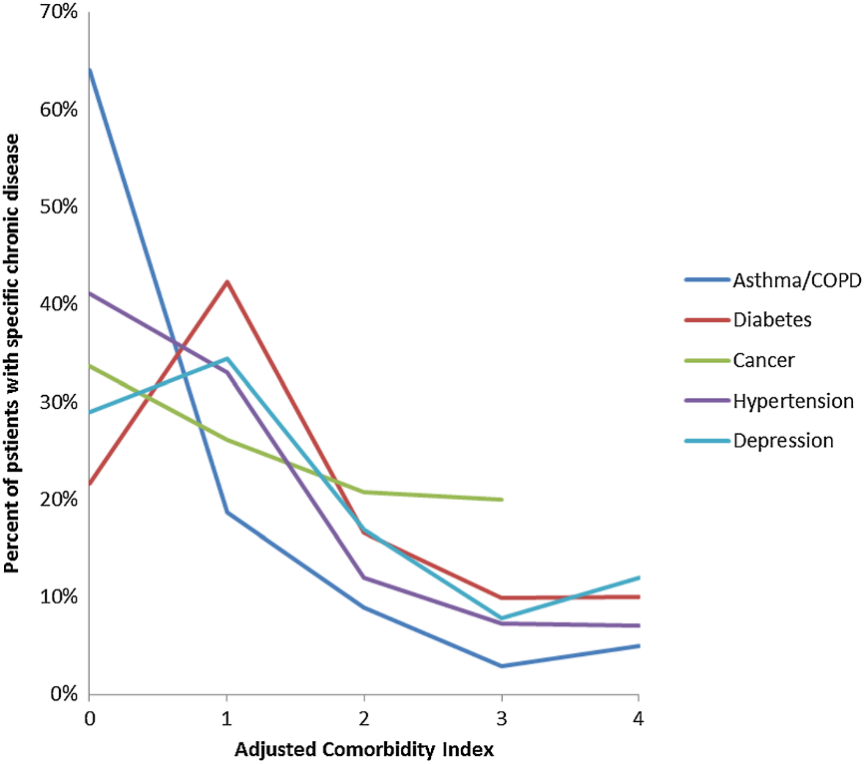
**The distribution of patients with specific chronic diseases according to their adjusted comorbidity score.**

Overall, as comorbidity increased, total costs increased (p < .01). Costs for each chronic condition did not differ at lower levels of comorbidity. Costs according to specific chronic illnesses were also evaluated. Figure 1 shows the costs for chronic diseases impacting at least 100 beneficiaries according to the adjusted level of comorbidity. It demonstrates that costs are more a function of the total burden of comorbidity than of any specific comorbid disease. Figure 2 shows the distribution of patients according to the adjusted comorbidity index; the vast majority of beneficiaries, regardless of specific chronic illness, had a comorbidity index of zero or one.

**Table 2:** **Beneficiaries and health care costs according to the Charlson comorbidity index**

Comorbidity	Number of beneficiaries	Percent of beneficiaries	Total costs	Percent of costs	Unadjusted cost per person	Adjusted cost per person
***Adults***						
0¥	1,203	54.2%	$886,611	30.9%	$737	$774
1	605	27.3%	$459,195	16.0%	$760	$741
2-3	137	6.2%	$299,208	10.4%	$2,184	$2,100
≥4	183	8.3%	$843,996	29.4%	$4,672	$4,553
All	2,128		$2,489,010			
***Children***						
0	2,089	87.2%	$607,899	62.1%	$291	$295
1	286	11.9%	$336,622	34.4%	$1,177	$1,152
2-3	5	0.2%	$6,250	0.6%	$1,251	$1,442
≥4	12	0.5%	$12,228	1.3%	$1,020	$111
All	2,392		$962,999			

¥ excludes 94 pregnant women 75 pregnant women with a comorbidity score of 1 had an average cost of $3,621, while the 16 women with a comorbidity score of 1 had costs of $5,470.

Adjusted costs are adjusted for age, gender, mental health, and addiction.

The comorbidity index and total visits were correlated for adults (R = .29) and children (R = .21); however; the comorbidity index did not increase until the number of visits exceeded 20.

### Pregnancy

The 94 women who were pregnant, including 4 adolescents, had a significantly increased cost of $4,104 ± $3,865 (p < .01). Of the pregnant women, 75 with a comorbidity score of 0 had an average cost of $3,621, while the 16 women with a comorbidity score of 1 had costs of $5,470. Only 3 pregnant women had scores of 3 or more; they had average costs of $8,902.

### Mental illness

Costs for the 190 patients with severe mental illness, including major depression, bipolar illness, and schizophrenia, were also significantly higher (p < .003). Of these patients with serious mental illnesses, 86% were adults, and 14% children; both groups had similar costs. Of the 100 patients who had an addiction, 93% were adults, and 7% children. Overall 3% of those with a comorbidity score of 0-1 had mental illness, vs. 15% of those with a score ≥2 (p < .01). Controlling for age, gender, comorbidity and pregnancy, patients with mental illness had almost double the costs than those without ($1,345 vs. $801).

### Hospitalization as a driver of costs

As shown in Table 3, the rate of hospitalization and repeated hospitalization increased with increasing comorbidity (p < .01 for both). Two-thirds of the patients hospitalized were adults, while one-third were children (p < .01). Patients who were hospitalized incurred mean costs of $8,996, while those not hospitalized had mean costs of $263 (p < .0001). Table 4 shows the average costs for patients according to comorbidity and total hospitalizations. Once comorbidity was taken into account, the hospitalization costs did not differ between children and adults (p < .7).

**Table 3:** **Percent of beneficiaries hospitalized according to comorbidity index**

Comorbidity	One hospitalization	Two or more hospitalizations	Any hospitalization
0-1	3.7%	1.0%	4.7%
2-3	13.3%	3.5%	16.8%
≥4	10.2%	6.6%	16.8%

**Table 4:** **Average cost according to number of hospitalizations and comorbidity index for all beneficiaries**

Comorbidity	No hospitalizations	One hospitalization	Two or more hospitalizations
0-1	$ 214	$ 5,511	$13,860
2-3	$ 926	$ 4,651	$24,713
≥4	$1,441	$14,468	$27,057

### Regression models

Results for the regression analysis for the two part model of log total costs are given in Table 5. For a pooled model of adults and children of log total costs, the comorbidity index was a key correlate of total costs (p < .01, 95% CI [.11, .14]). *Adults*: In addition to comorbidity (p < .01, 95% CI [.10, .14]), serious mental illness (p < .01, 95% CI [.06, .27]), and pregnancy (p < .01, 95% CI [.81, 1.07]) were also correlated with costs, while age, drug addiction and gender were not. The model with age, gender, comorbidity, mental illness, pregnancy, and drug/alcohol addiction explained 20% of the variance in total costs (R^2^ = .20) *Children:* In children, comorbidity ( p < .01, 95% CI [.10, .19]), serious mental illness (p < .01, 95% CI [.11, .53]), drug/alcohol addiction ( p < .03, 95% CI [.03, .81]), and pregnancy (p < .01, 95% CI [.89, 2.06]) were associated with log cost, however, age and gender were not significantly related to log total costs; the explained variance was 6% (R^2^ = .06).

**Table 5:** **Regression analysis for the two part model of log costs: Regression coefficients and t statistics**

	Adults and children combined	p	Adults	p	Child	p
Comorbidity	0.13	<.01	0.13	<.01	0.15	<.01
	(16.66)		(14.89)		(6.68)	
Age	0.00286	<.01	0.00059		-0.00466	
	(5.62)		(0.52)		(1.76)	
Female	0.00		0.04		-0.05	
	(0.00)		(1.22)		(1.91)	
Mental illness	0.19		0.16		0.32	
	(4.19)	<.01	(3.09)	<.01	(2.98)	<.01
Addiction	0.15		0.13		0.42	
	(2.46)	<.05	(1.91)		(2.13)	<.05
Pregnancy	1.02		0.95		1.48	
	(17.28)	<.01	(14.49)	<.01	(4.93)	<.01
Intercept	2.23		2.33		2.29	
	(120.44)	<.01	(40.47)	<.01	(83.58)	<.01
*R* ^2^	0.21		0.20		0.06	
*N*	3,427		1,881		1,546	

Observed log10 Total Cost regression coefficients from a two part regression model (equations (1) and (2) in the Appendix). The coefficients for the zero inflation part of the two part model are not displayed. T-statistics are in parentheses.

Quantile regression was used to evaluate predictors of the top 5% and 10% of cost; the results are presented in Table 6. The comorbidity index was significantly correlated with the top 5% and top 10% of costs for the pooled sample, as well as for adults and children separately. For the top 10%, pregnancy and serious mental illness were significantly associated with log total costs in the adult sample while age was negatively correlated with log total costs in the child sample.

**Table 6:** **Quantile regression analysis of correlates of the top 10% and top 5% of costs: Regression coefficients and t statistics**

	Top 10% combined		Top 5% combined		Top 10% of adults		Top 10% child	
	Adult and child	p	Adult and child	p		p		p
Comorbidity	0.19		0.21		0.18		0.33	
	(10.83)	<.01	(7.01)	<.01	(10.67)	<.01	(3.70)	<.01
Age	0.004		0.00089		0.00111		-0.02751	
	(3.33)	<.01	(0.45)		(0.49)		(2.58)	<.01
Female	-0.02		-0.05		-0.05		-0.12	
	(0.43)		(0.66)		(0.91)		(1.09)	
Mental illness	0.21		0.10		0.21		0.42	
	(1.86)		(0.53)		(1.98)	<.05	(0.96)	
Addiction	0.20		0.11		0.23		0.48	
	(1.37)		(0.45)		(1.71)		(0.60)	
Pregnant	0.93		0.77		0.87		0.98	
	(6.68)	<.01	(3.33)	<.01	(6.69)	<.01	(0.82)	
Intercept	2.83		3.24		3.03		3.43	
	(64.43)	<.01	(44.43)	<.01	(26.50)	<.01	(31.06)	<.01
*Pseudo R* ^*2*^	0.15		0.12		0.16		0.07	
*N*	3,427		3,427		1,881		1,546	

Observed log10 Total Cost regression coefficients from quantile regressions. The coefficients for the zero inflation part of the two part model are not displayed. T-statistics are in parentheses.

## Discussion

This study showed that costs for MMC beneficiaries were primarily driven by the aggregate burden of comorbid disease, not by individual chronic illnesses. This echoes the findings of other studies about multiple chronic diseases as drivers of cost [12–14]. Although comorbidity has been defined in some studies as a measure of ‘other diseases’ that can confound the outcomes of patients with a specific chronic illness, comorbidity is intrinsically a measure of the aggregate burden of disease.

For MMC programs, a critical question is how to control costs. To reduce costs, efforts have to focus on MMC beneficiaries most likely to incur high costs [15]. Some MMC efforts to decrease costs have targeted individuals who were high cost or high utilizers in the prior year [16–18] and others have focused on patients with specific chronic diseases [19–22]. However, most of these programs have not reduced costs [1, 22–24].

MMC cost reduction efforts have also focused on beneficiaries who are predicted to have high costs using claims based models [22–28]. Most efforts to reduce costs have not taken comorbidity into account [20, 22, 28]. However, in this analysis, the 8% of patients with a comorbidity burden of four or more had 30% of costs. This data shows that strategies focused on a single chronic disease per se cannot efficiently target patients with a high burden of comorbidity who have high costs.

Previous studies of Medicaid patients have had additional methodologic challenges [18]. Patients with such gaps in enrollment (churning in and out of Medicaid) have been shown to have highly variable costs [25].

### Limitations

The major limitation is that our analysis focused on claims and cost data for 6 months only. We could not ascertain whether costs differed by season, since we had only 6 months of data. It would have been preferable to have a full year of data. We did not evaluate patients who were cycling in and out of enrollment, who are known to have greater cost variability [25]. In addition, our analysis focused on non-disabled beneficiaries [29]. Another limitation is that the comorbidity index has not been validated for children or pregnant women. While costs are clearly higher among pregnant woman when compared to those with equivalent comorbidity burdens, the results for pregnant women have to be interpreted with caution because the subgroup is small.

Another limitation is that this study involves one MMC plan, based at one hospital in NYC. A recent comprehensive synthesis has pointed out that Medicaid is not a single program but a diverse group of state run programs, and that as a result it is hard to generalize about the impact of MMC on costs [1]. Thus, by this analysis alone, we cannot be certain that these findings would be applicable to other states or plans [1]. However, in analyses of two other populations, we have found a similar distribution of comorbidity and costs [5, 30].

## Conclusion

Patients with higher comorbidity incur higher costs, suggesting that high comorbidity patients may be a key target for cost savings in MMC. The comorbidity score provides a reproducible, valid method of identifying patients with multiple chronic diseases, who should be targets for cost-saving efforts.

## Appendix

### Statisical methods

In a two-part model, explanatory variables often play different roles in the two parts of the model. Since the nonzero expenditure data are always positive and heavily skewed to the right, they are modeled with a lognormal distribution. Let *Z*_*i*_ denote the observed total cost for the *i*th subject and ***X***_*i*_ represent a vector of subject specific characteristics. With inflated zero values, the observed costs are assumed to represent realizations of random variables that have probability distributions describable by a mixture of a point mass at zero and a continuous distribution. That is, *Z*_*i*_ = *I* [*Y*_1*i*_ > 0] *×* 10^*Y*2*i*^*,* where *Y*_*1i*_ and *Y*_*2i*_ represent two latent random variables, *I [A]* denotes the indicator of the event *A (=1 if A occurs and = 0 otherwise)*. Intuitively, *Y*_*1i*_ regulates when zero costs occurs and *Y*_*2i*_ is the logarithm (base 10) of non-zero costs. Zhang et al. [30] extend the two-part structure in Duan [31] to model the distributions of the two latent random variables *Y*_*1i*_ and *Y*_*2i*_. This model is at the individual level and adjusts for individual level characteristics. Specifically,
12

In these models, the covariates ***X***_*1i*_ and ***X***_*2i*_ can be different subsets of the complete set of observed characteristics, ***X***_*i*_ and the variance of *ϵ*_*1i*_ is set to one for identifiability. A consequence of Model 2 is that the actual nonzero costs are specified as a log-normally distributed random variable. An important feature of a log-normally distributed random variable model is the distribution is skewed and that the variance is exponentially increasing in the levels of the ***X***_*2i*_ [30]. Estimation for the models defined by (1) and (2) is carried out using maximum likelihood estimation.

In the regression models, we use Model 1 to model the zero cost and Model 2 to model the cost level. We use the comorbidity index age, gender, comorbidity, mental illness, pregnancy, and drug/alcohol addiction as explanatory variables. One often defines the log normal distribution in terms of the natural logarithm, rather than base 10 used here, but other logarithmic bases lead to the same family of distributions with rescaled parameters.

## Authors’ information

VD: Chief Medical Officer at MetroPlus Health Plan.

WM: CEO/CMO, Gotham Health, NYC Health and Hospitals Corporation.

## Abbreviations

MMC: Medicaid managed care.
